# Implementation of coordinated global serotype 2 oral poliovirus vaccine cessation: risks of inadvertent trivalent oral poliovirus vaccine use

**DOI:** 10.1186/s12879-016-1537-8

**Published:** 2016-06-01

**Authors:** Radboud J. Duintjer Tebbens, Lee M. Hampton, Kimberly M. Thompson

**Affiliations:** Kid Risk, Inc, 10524 Moss Park Rd., Ste. 204-364, Orlando, FL, 32832 USA; Global Immunization Division, Center for Global Health, Centers for Disease Control and Prevention, Atlanta, GA 30333 USA

**Keywords:** Polio, Eradication, Risk management, Oral poliovirus vaccine, Dynamic modeling, Vaccine-derived poliovirus

## Abstract

**Background:**

The endgame for polio eradication includes coordinated global cessation of oral poliovirus vaccine (OPV), starting with the cessation of vaccine containing OPV serotype 2 (OPV2) by switching all trivalent OPV (tOPV) to bivalent OPV (bOPV). The logistics associated with this global switch represent a significant undertaking, with some possibility of inadvertent tOPV use after the switch.

**Methods:**

We used a previously developed poliovirus transmission and OPV evolution model to explore the relationships between the extent of inadvertent tOPV use, the time after the switch of the inadvertent tOPV use and corresponding population immunity to serotype 2 poliovirus transmission, and the ability of the inadvertently introduced viruses to cause a serotype 2 circulating vaccine-derived poliovirus (cVDPV2) outbreak in a hypothetical population. We then estimated the minimum time until inadvertent tOPV use in a supplemental immunization activity (SIA) or in routine immunization (RI) can lead to a cVDPV2 outbreak in realistic populations with properties like those of northern India, northern Pakistan and Afghanistan, northern Nigeria, and Ukraine.

**Results:**

At low levels of inadvertent tOPV use, the minimum time after the switch for the inadvertent use to cause a cVDPV2 outbreak decreases sharply with increasing proportions of children inadvertently receiving tOPV. The minimum times until inadvertent tOPV use in an SIA or in RI can lead to a cVDPV2 outbreak varies widely among populations, with higher basic reproduction numbers, lower tOPV-induced population immunity to serotype 2 poliovirus transmission prior to the switch, and a lower proportion of transmission occurring via the oropharyngeal route all resulting in shorter times. In populations with the lowest expected immunity to serotype 2 poliovirus transmission after the switch, inadvertent tOPV use in an SIA leads to a cVDPV2 outbreak if it occurs as soon as 9 months after the switch with 0.5 % of children aged 0–4 years inadvertently receiving tOPV, and as short as 6 months after the switch with 10–20 % of children aged 0–1 years inadvertently receiving tOPV. In the same populations, inadvertent tOPV use in RI leads to a cVDPV2 outbreak if 0.5 % of OPV RI doses given use tOPV instead of bOPV for at least 20 months after the switch, with the minimum length of use dropping to at least 9 months if inadvertent tOPV use occurs in 50 % of OPV RI doses.

**Conclusions:**

Efforts to ensure timely and complete tOPV withdrawal at all levels, particularly from locations storing large amounts of tOPV, will help minimize risks associated with the tOPV-bOPV switch. Under-vaccinated populations with poor hygiene become at risk of a cVDPV2 outbreak in the event of inadvertent tOPV use the soonest after the tOPV-bOPV switch and therefore should represent priority areas to ensure tOPV withdrawal from all OPV stocks.

**Electronic supplementary material:**

The online version of this article (doi:10.1186/s12879-016-1537-8) contains supplementary material, which is available to authorized users.

## Background

Under current plans, use of oral poliovirus vaccine (OPV) will cease in a globally-coordinated, staged manner, starting with the withdrawal of all trivalent OPV (tOPV) containing serotypes 1, 2, and 3 live, attenuated polioviruses between April 17 and May 1, 2016 [1–3]. With eradication of serotype 2 wild polioviruses (WPVs) now certified [4], countries using tOPV at that time will switch to using bivalent OPV (bOPV), which contains only serotypes 1 and 3. This change in OPV use will end new infections with the serotype 2 attenuated viruses found in tOPV that can lead to serotype 2 vaccine-associated paralytic poliomyelitis and serotype 2 circulating vaccine-derived polioviruses (cVDPV2s). Although very rare, cVDPVs can emerge in communities with low vaccination coverage as a result of genetic changes that accumulate as the OPV viruses and their descendants replicate during continued person-to-person transmission. Due to the preferential use of bOPV in supplemental immunization activities (SIAs), cVDPV2s accounted for the vast majority of cVDPVs since 2006 [5–7]. The end of tOPV use will lead to decreasing population immunity to serotype 2 poliovirus transmission (i.e., defined as the collective level of protection to serotype 2 poliovirus transmission of all individuals in a population) as new birth cohorts accumulate with no exposure to serotype 2 live polioviruses [8, 9]. Decreasing population immunity to serotype 2 poliovirus transmission could allow post-switch use of tOPV to lead to reintroduction and subsequent ongoing transmission of serotype 2 polioviruses and eventually to the emergence of new cVDPV2s in an environment conducive to their further spread.

Prior modeling suggests that any outbreaks following OPV cessation of each serotype would require costly and aggressive response to control [10, 11]. Failure to mount an aggressive response would result in a high risk of eventual widespread propagation of the poliovirus serotype causing the outbreak to eventually spread to all areas that lack high population immunity after cessation of the corresponding OPV serotype. Moreover, of the two types of poliovirus vaccines currently available for outbreak response after OPV cessation, one type (i.e., monovalent OPVs that contain one live, attenuated poliovirus serotype) comes with a risk of creating new VDPVs, while the other (i.e., inactivated poliovirus vaccine (IPV) that contains type 1, 2, and 3 polioviruses that cannot replicate) does not significantly affect fecal-oral poliovirus transmission and remains untested in its ability to stop outbreaks in the developing world [10, 11]. Thus, outbreak prevention remains the most prudent approach to ensure a successful switch and subsequent endgame [11]. Prior studies emphasized the importance of several strategies to minimize the probability of an outbreak after the switch, including maximizing population immunity prior to the switch through the use of tOPV in supplemental immunization activities (SIAs) [8], identification and treatment of rare primary immunodeficient long-term poliovirus excretors with polio antiviral drugs [12], destruction or high-level bio-containment of polioviruses in laboratories and vaccine manufacturing sites [10, 13], use of IPV in routine immunization (RI) programs in all countries for several years [10], and ensuring a synchronous switch in all countries [14]. The last analysis showed how decreasing population immunity to transmission after the switch determines when different populations become vulnerable to circulation of imported serotype 2 OPV OPV2-related viruses and eventual cVDPV2 outbreaks in the event of a non-synchronous switch [14]. Assuming successful synchronization of the switch in all countries, potential inadvertent tOPV use after the switch may similarly lead to the development of cVDPV2 outbreaks because of the expected decrease in population immunity to serotype 2 poliovirus transmission. Thus, preventing inadvertent tOPV use after the switch represents an additional important risk management strategy to prevent post-switch cVDPV2 outbreaks.

Ensuring that countries withdraw tOPV from their cold chains and dispose of any remaining stocks soon after the switch to bOPV represents an important part of guaranteeing that tOPV is not inadvertently used after the switch. Nevertheless, the task of withdrawing tOPV from every health facility in all 155 countries that use or stockpile tOPV poses considerable logistical challenges [3]. For example, in India alone five tiers of facilities store vaccines as part of the cold chain, starting with four national government supply depots, progressing to 35 state vaccine stores, then 116 regional vaccine stores and 626 district vaccine stores, and finally 26,439 primary or community health centers [15]. Given the difficulties involved in withdrawing all tOPV from all facilities and then verifying compliance by all such facilities, estimates of the potential implications of the inadvertent use of variable amounts of tOPV after the switch represent important context for gauging the amount of resources warranted for tOPV withdrawal and disposal and for monitoring and verifying compliance of tOPV withdrawal.

This study complements a recent study of the vulnerability of populations to imported OPV2-related viruses in the event of a non-synchronous switch, which explored the reduction in population immunity to serotype 2 poliovirus transmission following the switch in different populations and the degree of reversion of OPV2-related viruses that circulate in countries that still use tOPV [14].

## Methods

We previously developed a differential equation-based poliovirus transmission and OPV evolution model (the DEB model) that tracks how individuals in a population move among numerous poliovirus-associated immunity states due to births, vaccination, exposure to polioviruses resulting from age-heterogeneous fecal-oral and oropharyngeal transmission, progression through infection stages, waning of immunity, and evolution of live, attenuated OPV to fully-reverted VDPVs [16, 17]. We calibrated the DEB model to determine a set of generic model inputs, constrained by ranges obtained during an expert literature review and elicitation process [6, 18, 19] that produces behavior consistent with the evidence about paralytic polio incidence, vaccine histories, age distributions of cases, serology, secondary OPV exposure, serotype differences, die-out of WPV, and emergence of cVDPVs or lack thereof in 10 distinct situations [16, 17, 20].

The evolution of poliovirus originating with OPV in the DEB model occurs as a result of reversion of the OPV parent virus strain given to vaccinees (stage 0) to 19 successive stages of OPV-related virus with increasing transmissibility (characterized by relative basic reproduction number (R_0_) compared to typical homotypic WPVs in the same setting) and neurovirulence (characterized by relative paralysis-to-infection ratio (PIR) compared to typical homotypic WPVs in the same setting). The model assumes that poliovirus in the last reversion stage (stage 19, i.e., fully-reverted VDPV) has the same R_0_ and PIR as typical homotypic WPVs in the same setting. In the DEB model, transmission of any live poliovirus (LPV, i.e., WPV or OPV-related virus in any individual reversion stage, including OPV parent virus strains and VDPVs) requires a minimum prevalence of 5 effective infections per million people (i.e., the transmission threshold), with 0 force-of-infection assumed for lower prevalence to simulate die-out in the deterministic DEB model. With ongoing OPV use, the prevalence of OPV parent virus (stage 0) typically remains above the transmission threshold so that some secondary OPV parent virus (stage 0) transmission occurs, which will generate more OPV parent virus (stage 0) infections. At the same time, a fraction of infections with the viruses descended from the OPV parent virus (stage 0) will evolve to the next reversion stage, potentially resulting in prevalence above the transmission threshold and generation of new infections in that reversion stage. Thus, the prevalence in any reversion stage after stage 0 depends on the prevalence in the preceding reversion stage and the force-of-infection in the reversion stage. The force-of-infection depends on the R_0_ of the reversion stage and population immunity to transmission. With high enough population immunity to transmission, each infection in the lower reversion stages generates fewer than one new infection on average (i.e., the mixing-adjusted net reproduction number (R_n_) is less than 1 [14, 21]), so that the prevalence in the higher reversion stages never exceeds the transmission threshold. However, with low population immunity to transmission and some level of OPV use, new infections in lower reversion stages generate enough new infections (i.e., R_n_ closer to or greater than 1) to sustain prevalence over the threshold in higher reversion stages. This can allow evolution to even higher reversion stages and eventual emergence of VDPV circulation (i.e., a cVDPV outbreak). While this characterization of OPV evolution using the transmission threshold merely approximates the true micro-dynamics and random events that play a role in real OPV evolution and cVDPV emergence, it accounts for the interplay between OPV use and population immunity [8] and has adequately reproduced cVDPV outbreaks in places like northwest Nigeria (serotype 2), Madura (Indonesia; serotype 1), Haiti (serotype 1), northern India (serotype 2) and lack of cVDPV outbreaks despite widespread OPV use in places like the USA, the Netherlands (following an outbreak in 1992–3), Israel, Tajikistan, Albania, Cuba, northwest Nigeria (serotypes 1 in and 3) and northern India (serotypes 1 and 3) [16, 17, 20].

In a prior analysis [14] we used the R_n_ of OPV2-related viruses in various reversion stages as a proxy measure of the vulnerability to widespread circulation following an importation of such a virus from a population that did not yet switch, with an R_n_ > 1 indicating a minimum condition for circulation. In this study, we focus on inadvertent tOPV use in a population that has already switched, which differs from point introductions in two ways that change the minimum R_n_ of OPV2-related virus needed for subsequent circulation and outbreaks. First, inadvertent tOPV use implies an introduction of OPV2 parent virus (stage 0) while importations may involve more reverted and thus relatively more transmissible OPV2-related virus that imply a potentially higher risk of circulation. Second, inadvertent tOPV use potentially involves large numbers of doses given to children in a short period of time, while importations represent point introductions. Inadvertent administration of a large number of tOPV doses implies some possibility that one of the doses by chance leads to a high degree of reversion through successive transmissions or mutations occurring in individual recipients [22]. The DEB model mimics this possible outcome because more inadvertent tOPV doses results in a higher prevalence of OPV parent virus (stage 0) and virus in subsequent stages. However, this may or may not lead to emergence of highly-reverted virus and eventual cVDPV2 outbreaks, depending on the preexisting population immunity to serotype 2 poliovirus transmission and the population immunity induced by the inadvertent tOPV use.

**Fig. 1:**
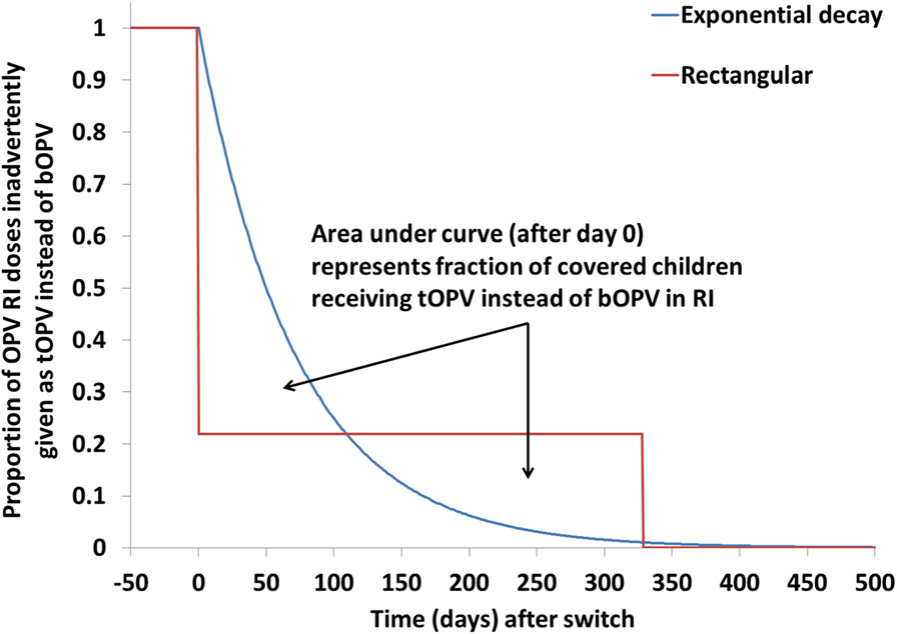
Modeled patterns of inadvertent trivalent oral poliovirus vaccine (tOPV) use in routine immunization (RI) (Analysis II)

We examine inadvertent tOPV use both in an SIA (Analysis I) and in routine immunization (RI) (Analysis II) and determine under which conditions such use can lead to a cVDPV2 outbreak. The analysis of inadvertent tOPV use in an SIA assesses the consequences of a one-time simultaneous administration of tOPV to children aged 0–4 years, while the analysis of inadvertent tOPV use in RI assesses the consequences of administering tOPV over time to children aged 0–1 years as they reach scheduled ages for OPV doses (approximated in the DEB model to occur as a single dose at birth (for countries that give a birth dose) and the cumulative effect of 3 non-birth doses at 3 months) [16]. For Analysis I, we focus on the interaction between the extent of inadvertent tOPV use in an SIA and the time after the switch when this occurs. For Analysis II, we consider different potential patterns of continued inadvertent tOPV use in RI (Fig. 1). A pattern of exponential decay corresponds to a scenario in which a population gradually uses up all tOPV in its RI supply chain until exhausted. For this scenario, we determine the minimum half-life (i.e., the time over which the extent of tOPV use decreases by a half) for which the inadvertent tOPV use in RI leads to a cVDPV2 outbreak. A rectangular pattern corresponds to a scenario in which some fraction of health centers in a population inadvertently continue to use tOPV for a period of time after the switch. For this scenario, we focus on the interaction between the extent of inadvertent tOPV use (i.e., the height of the rectangle) and its duration (i.e., the length of the rectangle).

All processes in the DEB model (e.g., vaccination, mixing and poliovirus transmission, die-out, OPV evolution, and outbreak detection) occur on a per-capita basis and consequently the model remains completely scalable. For example, post-switch inadvertent administration of 1,000 tOPV doses in a population of 10 million people produces 10 times higher absolute incidence of poliovirus infections and paralytic cases but exactly the same transmission and OPV evolution dynamics as inadvertent administration of 100 tOPV doses in a population of 1 million people with otherwise identical properties, including spatially-homogeneous mixing. Therefore, rather than specifying absolute numbers of inadvertent tOPV doses and population sizes, for Analysis I we express the extent of inadvertent tOPV use in an SIA in terms of the proportion of children aged 0–4 years in the population who inadvertently receive a dose of tOPV instead of bOPV during an SIA (i.e., the inadvertent tOPV SIA coverage). For Analysis II, we express the extent of inadvertent tOPV use in RI in terms of the proportion of OPV RI doses inadvertently given as tOPV instead of bOPV (i.e., the inadvertent tOPV RI proportion).

The left columns of Table 1 provide the properties of all populations in which we explored the implications of inadvertent tOPV use. For both Analysis I and II, we first examine inadvertent tOPV use after the switch in a hypothetical population with no seasonal variation in R_0_ values. We assume no seasonality to ensure a continuous decrease in the ability of polioviruses to transmit (i.e., the R_n_) as population immunity to serotype 2 poliovirus transmission decreases after the switch. This yields a theoretical minimum time and R_n_ until inadvertent tOPV use may lead to a cVDPV2 outbreak. We repeat the analysis for two different R_0_ values to demonstrate the impact of population-specific characteristics also without the complication of seasonality. In the DEB model, the R_0_ values of all serotypes and reversion stages depend directly on the R_0_ of serotype 1 wild poliovirus (WPV1), which we multiply by the appropriate serotype-specific relative R_0_ values (i.e., 0.9 and 0.75 for serotypes 2 and 3, respectively) [16, 17]. For brevity, we use the R_0_ of WPV1 as a general measure of the inherent transmissibility of polioviruses in any given population (i.e., instead of listing the values for each serotype).

**Table 1:** Populations modeled and tabulated selected results from Fig. 2 (Analysis I)

Population with properties like	Properties									Minimum time (years) since switch when inadvertent tOPV use in an SIA leads to a cVDPV2 outbreak for indicated inadvertent tOPV SIA coverage			
	Income level	R_0_^a^	p^oro^	tr	POL3	# tOPV SIAs (2015–2016)	TC	P_rm_	dt	0.1 %	0.5 %	1 %	Inadvertent tOPV coverage (given in parentheses) leading to shortest minimum time
Hypothetical population	Lower middle		0.3	0.6	0.3	4	0.8	0.7	1				
- High R_0_		13^b^								1.2	0.83	0.72	0.47 (0.15)
- Lower R_0_		10^b^								1.8	1.3	1.1	0.86 (0.15)
Northern India	Lower middle	13^c^	0.3	0.6		6			1				
- Under-vaccinated					0.3		0.8	0.7		1.2	0.88	0.82	0.65 (0.2)
- General					0.6		0.95	0.5		1.7	0.95	0.87	0.71 (0.2)
Northern Pakistan/Afghanistan	Low	11^c^	0.3	0.65					3				
- Under-vaccinated					0.1	5	0.35	0.95		0.94	0.76	0.7	0.52 (0.15)
- General					0.6	4	0.8	0.7		1.8	0.99	0.91	0.75 (0.2)
Northern Nigeria	Lower middle	8^d^	0.3	0.7		7							
- Under-vaccinated					0.05		0.15	0.95	3	1.5	0.8	0.7	0.51 (0.1)
- General					3		0.8	0.7	2	1.9	1.6	1.5	1.3 (0.1)
Ukraine	Upper middle	6^e^	0.8	0.74		0	0.8	0.7	3				
- Under-vaccinated					0.3^f^					2.9	1.8	0.93	0.82 (0.1)
- General					0.7^f^					32	12	8.9	6.7 (0.1)

*Abbreviations*: *cVDPV2* serotype 2 circulating vaccine-derived poliovirus, *RI* routine immunization, *SIA* supplemental immunization activity, *tOPV* trivalent oral poliovirus vaccine, *WPV* wild poliovirus

*Model input symbols*: [8, 16] *R*_*0*_ average annual basic reproduction number for WPV of serotype 1, *tr* take rate of serotype 2 tOPV, *POL3* RI coverage with 3 or more non-birth doses, *TC* true coverage of each SIA, *p* ^*oro*^ proportion of transmissions via oropharyngeal route, *dt* detection threshold (cumulative paralytic polio cases per 10 million people until outbreak detection occurs)

^a^The model uses R_0_ for serotype 1 WPV to characterize variability in subpopulations; R_0_ for serotype 2 WPV equals 0.9 times the values shown in this column

^b^No seasonality

^c^Seasonal amplitude in R_0_ of 20 % with peak on 180^th^ day of each year

^d^Seasonal amplitude in R_0_ of 10 % with peak on 120^th^ day of each year

^e^Seasonal amplitude in R_0_ of 40 % with peak on 180^th^ day of each year

^f^Assume POL3 = 90 % prior to 2010

In real populations, R_0_ varies seasonally, which means that the time during the year of inadvertent tOPV use influences the risk that the use leads to a cVDPV2 outbreak. As with the earlier study [14], we adopt the properties from selected populations included in an integrated global model for long-term poliovirus risk management (i.e., the global model) [10] as representative of real populations to serve as examples of when inadvertent tOPV use after the switch may lead to a cVDPV2 outbreak in realistic populations.. The properties (Table 1) include the R_0_ value for WPV1 and its seasonal variation through sinusoidal variation of the R_0_ values according to given amplitude and annual peak day [16] and thus the results of our model for the realistic populations account for the effect of seasonality. Other properties include the proportion of transmissions occurring via the oropharyngeal route (p^oro^, which strongly influences the ability of IPV alone to provide population immunity to transmission, since IPV provides good protection from oropharyngeal excretion but little protection from fecal excretion [18, 23]), the take rate of the serotype 2 component of tOPV (tr), the quality of acute flaccid paralysis (i.e., modeled using a detection threshold (dt) of cumulative paralytic cases that need to occur for the surveillance system to detect an outbreak), and a simplified vaccination history (i.e., RI coverage with 3 or more poliovirus vaccine doses (POL3) and any changes in RI vaccines, historic SIA frequency and SIA vaccine choices, and SIA quality). To determine the demographic profilesfor the populations, we directly adopt the average birth rates and age-specific mortality rates of populations from the global model, which differentiates by income level and polio vaccine use as of 2013 (i.e., OPV-only, IPV-only, or IPV/OPV) [10]. We focus on realistic populations with properties like those of northern India, northern Pakistan and Afghanistan, northern Nigeria, and Ukraine because they represent high-risk settings due to high R_0_ values, the presence of under-vaccinated subpopulations, and/or recent disruptions in immunization programs. The population with properties like those of Ukraine includes some adaptations relative to the global model assumptions for this part of the world to account for specifics of this example, including POL3 of 90 % before 2010 followed by a drop to 70 % (general population) or 30 % (under-vaccinated population), a series of SIAs in the 1990s, and adoption of an IPV/OPV sequential schedule (i.e., 2 doses of IPV followed by 2 doses of tOPV) in 2005 [14]. All other populations assume introduction of a single IPV dose on January 1, 2015. Although the realistic populations in Table 1 reflect assumptions representative of true settings based on prior work [17, 19], the use of simplified vaccination histories from the global model [10] means that the models will not exactly reproduce the paralytic polio incidence, WPV elimination, and past cVDPV outbreaks in those populations. However, we believe that the model arrives at accurate estimates of levels of population immunity to transmission at the time of the switch and beyond in real populations because the assumptions about R_0_, RI coverage, SIA frequency and quality, and other properties remain similar to those of real populations [10, 16, 17, 20, 21, 24–26].

For the hypothetical population in Analysis I, we vary the inadvertent tOPV SIA coverage from 0.1 to 99 % to explore the full relationship between the inadvertent tOPV SIA coverage and the minimum time until the inadvertent tOPV use in an SIA leads to a cVDPV2 outbreak. After determining from the hypothetical population the inadvertent SIA coverage above which the risk of an eventual cVDPV2 outbreak decreases due to the immunity provided by the inadvertent tOPV use in an SIA, for the realistic populations we estimate the minimum time until inadvertent tOPV use in an SIA leads to a cVDPV2 outbreak for inadvertent tOPV SIA coverage of 0.1, 0.5, 1, 5, 10, 15, 20, and 25 %. We determine the minimum time until the inadvertent tOPV use in an SIA leads to a cVDPV2 outbreak in the model by iteratively varying the first day of the 5-day SIA until detection of an outbreak, based on the population-specific detection thresholds (Table 1). Similarly, for Analysis II, we vary the inadvertent tOPV RI proportion from 0.1 to 100 % for the hypothetical population to determine the inadvertent tOPV RI proportion above which the risk of an eventual cVDPV2 outbreak decreases due to the immunity provided by the inadvertent tOPV use in RI. For the realistic populations, we consider inadvertent tOPV RI proportions of 0.5, 1, 5, 10, 15, 20, 25, and 50 % and use the same iterative approach to determine the shortest duration of inadvertent tOPV use that leads to an eventual cVDPV2 outbreak in the model. If the shortest duration occurs for an inadvertent tOPV RI proportion of 25 or 50 %, we also run values of 30, 35, 40, and 45 % to determine the approximate shortest duration in that range.

## Results

**Fig. 2:**
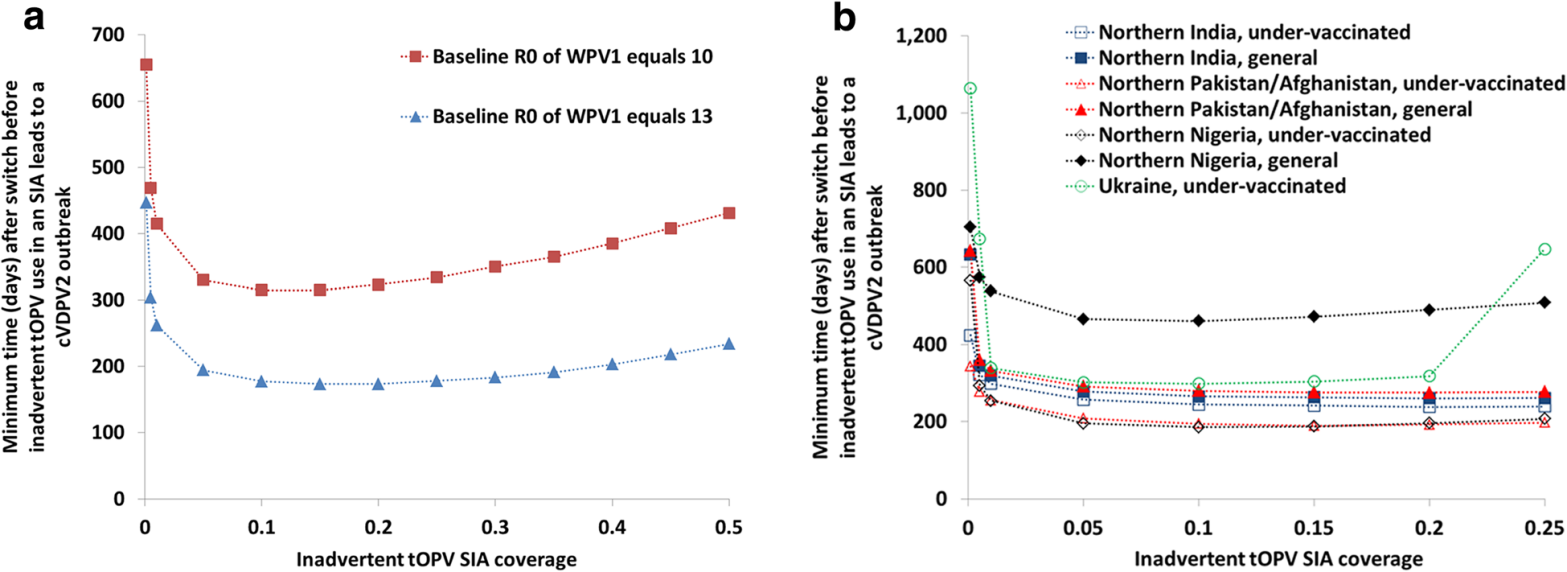
Minimum time until inadvertent trivalent oral poliovirus vaccine (tOPV) use in a supplemental immunization activity (SIA) leads to a serotype 2 vaccine-derived poliovirus (cVDPV2) outbreak (Analysis I) **a** in a hypothetical population with no seasonality in the basic reproduction number (R_0_) **b** in realistic populations with seasonality

Figure 2 shows the results of Analysis I and illustrates the relationship between the inadvertent tOPV SIA coverage and the minimum time since the switch for the inadvertent tOPV use to lead to a cVDPV2 outbreak. Table 1 includes selected results from Fig. 2 in tabulated form. Inadvertent administration of tOPV to a very small proportion of children (i.e., 0.1 %) in a hypothetical population with no seasonality only leads to a cVDPV2 outbreak if it occurs more than a year after the switch, when population immunity to serotype 2 poliovirus transmission has decreased significantly (Fig. 2a). Once population immunity to serotype 2 poliovirus transmission decreases sufficiently, some transmission of OPV parent virus (stage 0) can occur, which leads to reversion to subsequent stages of OPV2-related virus and circulation of those viruses (i.e., prevalence exceeding the transmission threshold), until ultimately a reversion stage that can self-amplify (i.e., R_n_ exceeds 1) begins to circulate. Once this occurs, circulation and reversion continues and a cVDPV2 outbreak will occur. We observed that inadvertent administration of tOPV to 0.1 % of children aged 0–4 years in an SIA leads to a cVDPV2 outbreak if it occurs when the R_n_ of OPV2 (stage 0) in the model exceeds approximately 0.85. Thus, while the R_n_ of OPV2 parent virus (stage 0) remains less than 1 at the time of the inadvertent tOPV use, even a small fraction receiving an inadvertent tOPV dose can generate enough reversion to start circulation of higher reversion stages. The R_n_ for OPV2 parent virus (stage 0) at the time of the switch depends in part on the assumed R_0_ (i.e., R_n_ equals 0.44 for an R_0_ of 13 and R_n_ equals 0.49 for an R_0_ of 10), and consequently the time until R_n_ reaches a high enough value to allow a cVDPV2 outbreak following inadvertent tOPV use depends on the assumed R_0_. Assuming an R_0_ of 13, R_n_ for OPV2 parent virus (stage 0) first exceeds 0.85 447 days after the switch in the hypothetical population, while for an R_0_ of 10, this only occurs after 655 days (Fig. 2a).

As we increase the inadvertent tOPV SIA coverage, the prevalence in subsequent reversion stages due to reversion and secondary transmission also increases. Therefore, an eventual cVDPV2 outbreak can occur for lower R_n_ values of OPV2 parent virus (stage 0) and thus sooner after the switch as larger proportions of children receive tOPV simultaneously after the switch. For example, with inadvertent tOPV SIA coverage of 1 %, a cVDPV2 outbreak can occur when the R_n_ of OPV2 parent virus (stage 0) exceeds approximately 0.65 at the time of the inadvertent tOPV SIA, corresponding to 262 or 415 days after the switch for an assumed R_0_ of WPV1 of 13 or 10, respectively. As we further increase the inadvertent tOPV SIA coverage, the time until inadvertent tOPV use in an SIA can lead to a cVDPV2 outbreak further decreases. However, at inadvertent tOPV SIA coverage of around 15 %, the inadvertent tOPV use in an SIA begins to confer sufficient population immunity to serotype 2 poliovirus transmission to reduce R_n_ and thus make it more difficult for a cVDPV2 to emerge. This represents the worst-case scenario of inadvertent tOPV use in an SIA in a spatially-homogenously mixing population, with greater inadvertent tOPV SIA coverage less likely to lead to a cVDPV2 outbreak (i.e., higher R_n_ of OPV2 needed and longer time since the switch). With inadvertent tOPV SIA coverage of 15 %, the shortest time since the switch for the inadvertent tOPV use to lead to a cVDPV2 outbreak equals 173 or 315 days after the switch for an assumed R_0_ of WPV1 of 13 or 10, respectively. For context, assuming the population size of approximately 10 million people as of 2013 used in the global model [10], the worst-case inadvertent tOPV SIA coverage of 15 % corresponds to approximately 165,000 children in this population, while inadvertent tOPV SIA coverage of 0.1 % corresponds to approximately 1,100 children. At inadvertent tOPV SIA coverage of 99 %, the minimum time after the switch for this to lead to a cVDPV2 outbreak extends to 651 and 875 days when the R_0_ of WPV1 equals 13 and 10, respectively (not shown in Fig. 2).

Figure 2b explores the minimum time until inadvertent tOPV use can lead to a cVDPV2 outbreak as a function of the inadvertent tOPV SIA coverage for realistic populations that include seasonal variation in poliovirus transmissibility, so that the timing of the inadvertent tOPV use relative to the seasonal fluctuations in R_0_ values affects the subsequent emergence of cVDPV2s. All populations exhibit a sharp drop in the minimum time until a cVDPV2 outbreak can occur with increasing inadvertent tOPV SIA coverage, with the shortest time for 10–20 % inadvertent tOPV SIA coverage, and an increase in the minimum time for higher coverage values, similar to Fig. 2a. However, the reality of seasonality affects the shape of the curves. For example, due to the assumed strong seasonality in the population with properties like those of the under-vaccinated subpopulation in the Ukraine (Table 1), increasing the inadvertent tOPV SIA coverage from 0.1 to 0.5 % in this population decreases the minimum time until inadvertent tOPV use can lead to a cVDPV2 outbreak by over one year because the higher initial prevalence of tOPV allows the cVDPV2 to emerge one high season earlier. The shortest times until inadvertent tOPV use in an SIA can lead to a cVDPV2 outbreak occur in under-vaccinated populations with properties like those of northern Pakistan and Afghanistan and northern Nigeria, which coincide with the areas in which interruption of WPV transmission proved most challenging and in which very low RI coverage with IPV will provide almost no population immunity to serotype 2 poliovirus transmission after the switch. In historically under-vaccinated populations in northern India, immunization quality improved significantly during the last stages of eradication, which if sustained will provide high population immunity to serotype 2 poliovirus transmission at the time of the switch and will thus extend the time until inadvertent tOPV use in a SIA can lead to a cVDPV2 outbreak, despite the high R_0_ values in northern India (Table 1).

Figure 2b shows a significantly lower risk in general populations compared to under-vaccinated populations due to the expected higher population immunity to serotype 2 poliovirus transmission in the general populations at the time of the switch (i.e., higher RI coverage and SIA quality, as shown in Table 1). For the population with properties like those of Ukraine, the difference between the general and under-vaccinated populations remains even more impressive, with the general population (i.e., with 70 % RI coverage) sustaining high enough population immunity to serotype 2 poliovirus transmission using IPV/bOPV or IPV-only schedules to prevent cVDPV2 outbreaks following inadvertent tOPV use for 7 years or more (not shown in Fig. 2 due to the choice of y-axis scale, but results included in Table 1). The ability of an IPV-only schedule to sustain high population immunity to serotype 2 poliovirus transmission in this population with properties like those of Ukraine comes from: (1) the higher relative proportion of transmissions occurring via the oropharyngeal route, (2) the lower absolute transmissibility of polioviruses in this setting (i.e., R_0_ of WPV1), and (3) the assumed use of a RI schedule that includes at least 2 IPV doses for upper middle-income countries (i.e., instead of 1 assumed for low- and lower middle-income populations). Despite these advantages, an under-vaccinated population with RI coverage of only 30 % but otherwise similar properties can generate a cVDPV2 outbreak following inadvertent tOPV use almost as quickly after the switch as general populations in northern India, northern Nigeria, and northern Pakistan and Afghanistan.

**Fig. 3:**
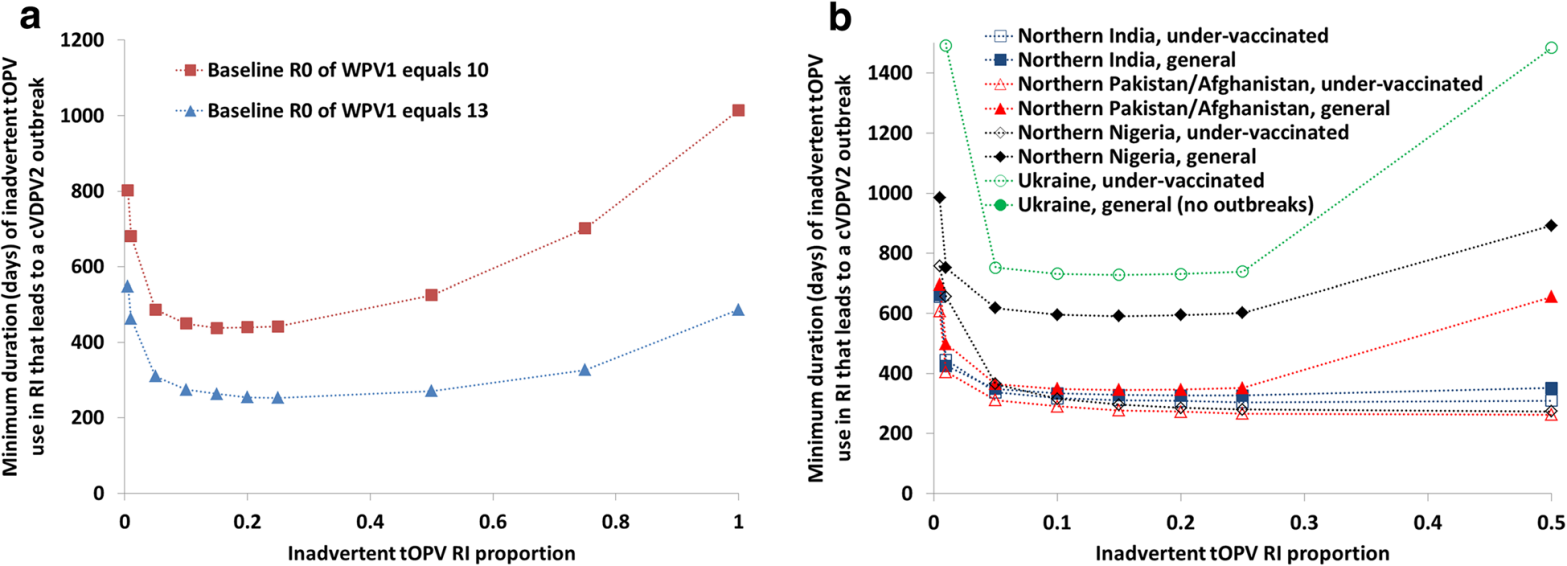
Minimum time until inadvertent trivalent oral poliovirus vaccine (tOPV) use in routine immunization (RI) leads to a serotype 2 circulating vaccine-derived poliovirus (cVDPV2) outbreak (Analysis II, rectangular pattern) **a** in a hypothetical population with no seasonality in the basic reproduction number (R_0_) **b** in realistic populations with seasonality

Table 2 and Fig. 3 show the results of Analysis II of inadvertent tOPV use in RI. In the hypothetical population without seasonality and an R_0_ of WPV1 of 13, gradually using up tOPV in the supply chain results in a cVDPV2 outbreak if the half-life (i.e., the time over which the extent of tOPV use decreases by a half) corresponding to this exponential decay process equals 0.16 years (2 months) or more (Table 2) (the Additional file 1 shows the kinetics of prevalence and evolution of OPV2-derived viruses for the exponential decay pattern in this population). We found similar minimum half-lives in populations with properties like those of northern India due to their high R_0_ values and resulting rapid drop in population immunity to serotype 2 poliovirus transmission after the switch, and in the under-vaccinated populations with properties like those of northern Pakistan and Afghanistan due to their low population immunity to serotype 2 poliovirus transmission at the time of the switch and relatively high R_0_ values (Table 1). Lower R_0_ values (e.g. in the hypothetical population with lower R_0_, and populations like Ukraine) result in longer minimum half-lives for an exponential decay in inadvertent tOPV RI use to lead to a cVDPV2 outbreak.

**Table 2:** Tabulated selected results of Analysis II from Fig. 3 and with the exponential decay scenario

Population with properties like^a^	Minimum half-life (years) before exponential decay in inadvertent tOPV use in RI leads to a cVDPV2 outbreak	Minimum duration (years) of rectangular inadvertent tOPV use in RI after the switch that leads to a cVDPV2 outbreak, for indicated inadvertent tOPV RI proportion			
		0.5 %	1 %	5 %	Proportion (given in parentheses) leading to shortest minimum duration
Hypothetical population					
- High R_0_	0.16	1.5	1.3	0.85	0.69 (0.25)
- Lower R_0_	0.27	2.2	1.9	1.3	1.2 (0.15)
Northern India,					
- Under-vaccinated	0.16	1.8	1.2	0.92	0.83 (0.25)
- General	0.17	1.8	1.2	0.96	0.89 (0.2)
Northern Pakistan/Afghanistan					
- Under-vaccinated	0.15	1.7	1.1	0.85	0.72 (0.5)
- General	0.22	1.9	1.4	1.0	0.95 (0.15)
Northern Nigeria					
- Under-vaccinated	0.22	2.1	1.8	1.0	0.75 (0.4)
- General	0.32	2.7	2.1	1.7	1.6 (0.15)
Ukraine					
- Under-vaccinated	0.81	2.7	2.1	1.7	1.6 (0.15)
- General	-^b^	-^c^	-^c^	-^c^	-^c^

*Abbreviations*: *cVDPV2* serotype 2 circulating vaccine-derived poliovirus, *RI* routine immunization, *tOPV* trivalent oral poliovirus vaccine

^a^See Table 1 for assumed properties for each population

^b^We observed either die-out of OPV2-related virus (for half-lives below approximately 4 years) or continued low-level circulation until the end of the analytical time horizon (i.e., 2053) (for longer half-lives)

^c^No cVDPV2 outbreak occurred for durations up to and including the last year of the analytical time horizon, although after > 15 years of inadvertent tOPV use in RI, the cumulative incidence of vaccine-associated paralytic poliomyelitis exceeded the detection threshold

Figure 3a illustrates the relationship between the inadvertent tOPV RI proportion and its duration in the event of continued inadvertent tOPV use in some fraction of RI (rectangular pattern) in a hypothetical population and Fig. 3b shows the results for realistic populations. Figure 3a reveals generally similar patterns as with inadvertent tOPV use in an SIA (Fig. 2). However, because the absolute RI coverage varies between the populations (Table 1), the shortest durations occur for different inadvertent tOPV RI proportions in each realistic population, and thus the shapes of the curves in Fig. 3b differ somewhat. For example, in the general population with properties like those of northern Pakistan and Afghanistan (with POL3 coverage of 60 %), an inadvertent tOPV RI proportion of 50 % represents much more tOPV use and thus provides much more population immunity to serotype 2 poliovirus transmission than an inadvertent tOPV RI proportion of 50 % in the under-vaccinated population with properties like those of northern Pakistan and Afghanistan (with POL3 coverage of 10 %). Consequently, the minimum duration of inadvertent tOPV use in RI after the switch for a cVDPV2 outbreak increases much faster between 25 and 50 % RI coverage in the general population than in the under-vaccinated population with properties like those of northern Pakistan and Afghanistan. In the under-vaccinated population with properties like those of Ukraine, the minimum duration of inadvertent tOPV use in RI after the switch that leads to a cVDPV2 outbreak remains substantially longer due to the population properties discussed in the context of Fig. 2b. With the higher RI coverage of the general population of 70 %, no duration of inadvertent tOPV use in RI leads to a cVDPV2 outbreak, although after multiple years the inadvertent tOPV use in RI would result in detection of serotype 2 VAPP cases (depending on the inadvertent tOPV RI proportion) and an increasing risk of exporting OPV2-related viruses that can circulate in other populations at some point in time after the switch [14]. Overall, continued inadvertent tOPV use in RI leads to a cVDPV2 outbreak somewhat later than inadvertent tOPV use in an SIA.

## Discussion

This study quantifies the minimum time after the tOPV-bOPV switch until inadvertent tOPV use may cause a cVDPV2 outbreak, assuming the recommended introduction of one IPV dose prior to the switch. Due to the kinetics of inadvertent tOPV introductions and OPV evolution, the results depend strongly on the proportion of children in a spatially-homogenously mixing population that inadvertently receive tOPV. Moreover, different populations experience very different times until inadvertent tOPV use may cause a cVDPV2 outbreak depending on their properties. Generally, higher basic reproduction numbers, lower tOPV-induced population immunity to serotype 2 poliovirus transmission at the time of the switch, and a lower proportion of transmission occurring via the oropharyngeal route all result in shorter times until inadvertent tOPV use in an SIA can lead to a cVDPV2 outbreak. With the exception of the general population with properties like Ukraine, which can maintain high enough population immunity to serotype 2 poliovirus transmission for many years using an IPV-only schedule, the modeled realistic populations represent some of the populations at highest risk of a cVDPV2 outbreak following inadvertent tOPV use. Thus, those populations and particularly their under-vaccinated subpopulations should warrant high scrutiny to ensure complete tOPV withdrawal at the time of the switch. While we did not model inadvertent tOPV use in all global populations, we do not expect most populations with good vaccination programs and relatively low R_0_ values to be able to generate cVDPV2 outbreaks following inadvertent tOPV use within a year of the switch. However, additional populations probably exist with poorly performing vaccinations programs (e.g., parts of sub-Saharan Africa, countries with areas of social unrest) and/or high poliovirus transmissibility (Bangladesh, parts of sub-Saharan Africa, rest of India, Pakistan, and Nigeria) that could experience a cVDPV2 outbreak following inadvertent use of large amounts of tOPV as soon as 6 months after the switch. In all populations, inadvertent use of very small amounts of tOPV seems unlikely to lead to a cVDPV2 outbreak for at least 1 year after the tOPV-bOPV switch.

The pattern of inadvertent tOPV use also affects the potential for a resulting outbreak. If inadvertent tOPV use occurs during an SIA, then this could lead to a cVDPV2 outbreak as soon as 6 months after the switch in the worst-case scenario among the populations we analyzed. If inadvertent tOPV use continues to occur in RI while gradually decreasing at a constant rate (e.g., exponential decay), then this could lead to a cVDPV2 outbreak if the extent of inadvertent tOPV use decreases by half every 2 months or more in the worst-case scenario. If inadvertent tOPV use occurs in RI at a constant low level (i.e., rectangular pattern) then this may lead to a cVDPV2 outbreak if it continues for at least 9 months after the switch in the worst-case scenario.

The reality that inadvertent tOPV use can lead to a cVDPV2 outbreak within a year of the switch in some populations despite IPV introduction prior to the switch supports the current policy of destroying all tOPV stocks at the time of the switch rather than using those stocks after the switch. Given limited global IPV supply, countries may be tempted to use any left-over tOPV stocks after the switch if they do not have IPV in order to provide vaccine recipients with immunity to serotype 2 poliovirus infections. However, doing so would result in a risk of causing cVDPV2 outbreaks after the switch. Due to the great variability in when countries would introduce IPV and the size of national stocks of tOPV, countries that continued to use tOPV until they introduced IPV or exhausted their tOPV stocks would likely stop using tOPV at very different times, allowing OPV2-related viruses to spread from countries continuing to use tOPV to countries in which population immunity to serotype 2 poliovirus transmission has declined following cessation of tOPV use. Such OPV2-related viruses could subsequently evolve into cVDPV2s, leading to cVDPV2 outbreaks [14]. Thus, countries should plan to either use tOPV in their supply chains prior to and not after the switch or to dispose of tOPV promptly after the switch. Any supplies of tOPV remaining at manufacturers at the time of the switch could potentially go into an outbreak response stockpile and find use as the preferred outbreak response vaccine for some countries simultaneously responding to a cVDPV2 and WPV1 during the time period between the tOPV-bOPV switch and the withdrawal of all types of OPV [11].

Our analysis relied on prior models [10, 14, 16] whose limitations also apply to this analysis. Specifically, the DEB model does not account for micro-level dynamics and random events that play a role in cVDPV2 emergences. The choice of the number of stages for OPV evolution influences the flows between reversion stages and thus when prevalence in an individual reversion stage drops below the transmission threshold due to transitions between reversion stages. Similarly, the multi-stage infection process with variable infectiousness for each infection stage [16] affects the kinetics of prevalence and die-out following an inadvertent tOPV release. Thus, as with all models the choice of model structure may affect the results, and we rely on a previously developed and calibrated model structure [10, 14, 16]. Future research may determine the importance of these assumptions and how they impact the findings. Furthermore, the realistic situations we modeled simplified the true vaccination histories in those settings and thus do not necessarily reflect the exact current conditions in those populations, although we believe they represent reasonable approximations of high-risk populations that exist in the real world. The results of the analyses also depend on the vaccination policies shortly before and during the tOPV-bOPV switch. For example, they assume well-implemented tOPV intensification in all countries that need to supplement their RI with SIAs before the switch [10, 27]. Failure to do so will not only lead to emergence of indigenous cVDPV2s after the switch in some populations [10, 27] and increase the risk of cVDPV2s in the event of a non-synchronous switch [14], but will also reduce the time until inadvertent tOPV use can lead to a cVDPV outbreak. Conversely, outbreak response activities in Ukraine [28] may effectively increase population immunity to serotype 2 poliovirus transmission and increase the time until inadvertent tOPV use can lead to a cVDPV outbreak. Finally, we did not model all populations or explore the potential effect of reversed seasonality in the Southern Hemisphere, which may increase or decrease the minimum time until inadvertent tOPV use can lead to a cVDPV2 outbreak.

## Conclusions

Efforts to ensure timely and complete withdrawal of tOPV at all levels, particularly from locations storing large amounts of tOPV, will help to minimize risks associated with the tOPV-bOPV switch. Under-vaccinated populations with poor hygiene become at risk of a cVDPV2 outbreak following inadvertent tOPV use the soonest after the tOPV-bOPV switch and therefore should represent priority areas to ensure tOPV withdrawal from all OPV stocks.

## Additional file

Additional file 1: Figure S1. Kinetics of prevalence and evolution of OPV2-derived viruses for the exponential decay pattern in the hypothetical population with a baseline R0 of WPV1 of 13. (EPI* = transmission threshold of effective proportion infectious below which the force-of infection becomes 0 in the differential equation-based model [15]). (PDF 1920 kb)

## Abbreviations

bOPV: bivalent oral poliovirus vaccine
cVDPV(2): circulating VDPV (serotype 2)
DEB: differential equation-based
IPV: inactivated poliovirus vaccine
LPV: live poliovirus
OPV(2): oral poliovirus vaccine (serotype 2 component)
R_0_: basic reproduction number
RI: routine immunization
R_n_: mixing-adjusted net reproduction number
SIA: supplemental immunization activity
tOPV: trivalent oral poliovirus vaccine
VDPV: vaccine-derived poliovirus
WPV(1): wild poliovirus (serotype 1)
