# Supplementary material for: Implementation of coordinated global serotype 2 oral poliovirus vaccine cessation: risks of inadvertent trivalent oral poliovirus vaccine use
Source: BMC Infect Dis. 2016 Jun 1;16:237. doi: 10.1186/s12879-016-1537-8 (PMC4888482; doi:10.1186/s12879-016-1537-8)

APPENDIX

Figure A1: Kinetics of prevalence and evolution of OPV2-derived viruses for the exponential decay pattern in the hypothetical population with a baseline R_0 of WPV1 of 13. (EPI* = transmission threshold of effective proportion infectious below which the force-of-infection becomes 0 in the differential equation-based model [15]).

(a) No inadvertent tOPV use

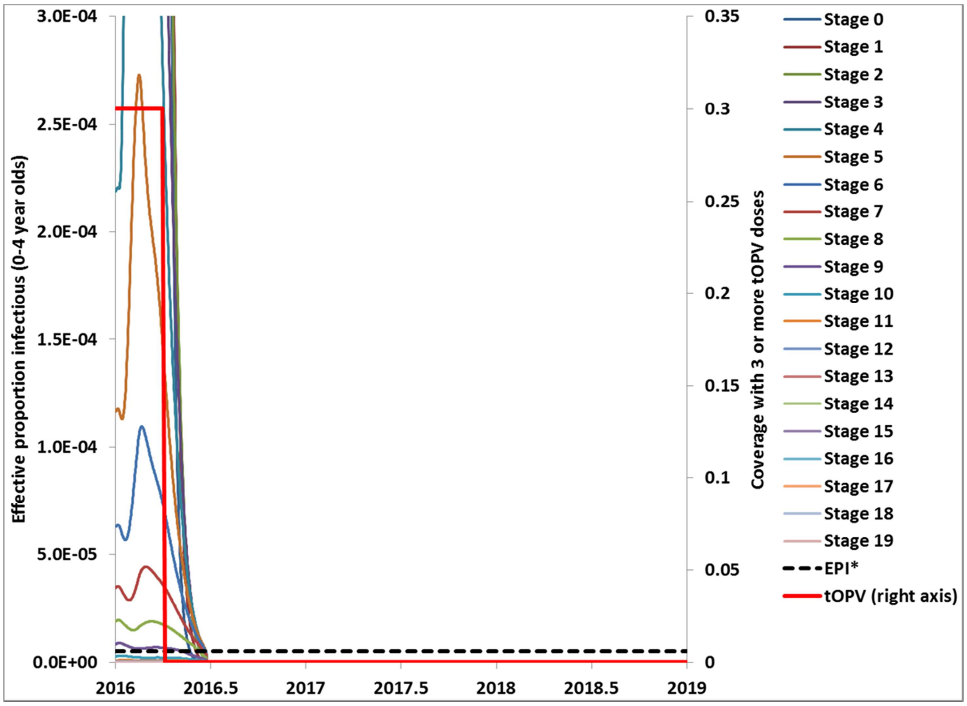

(b) Half-life of 10 days

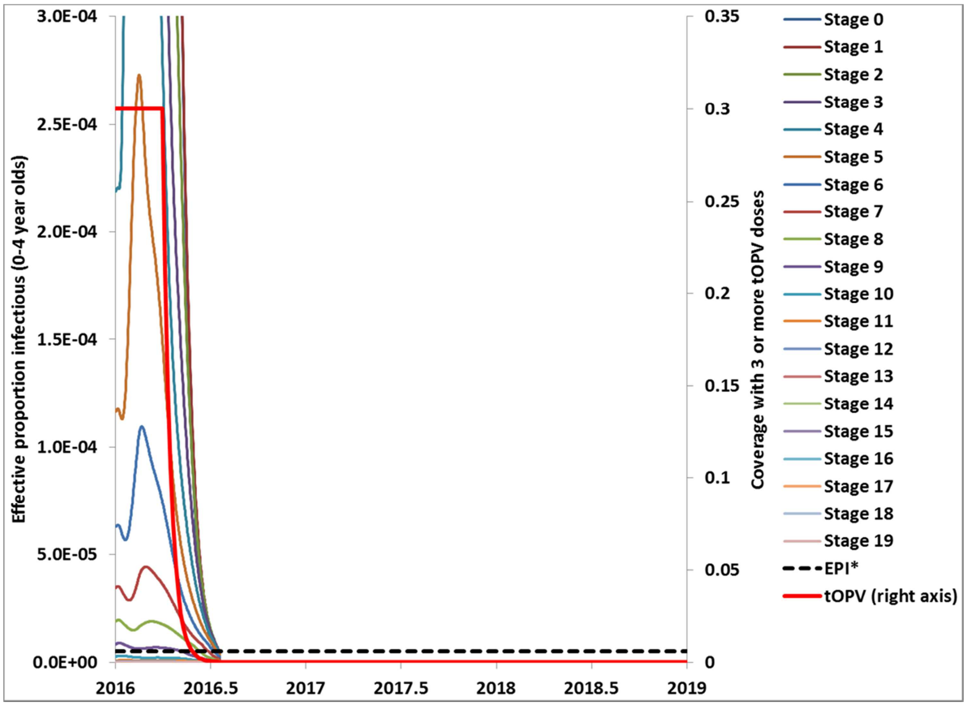

(c) Half-life of 20 days

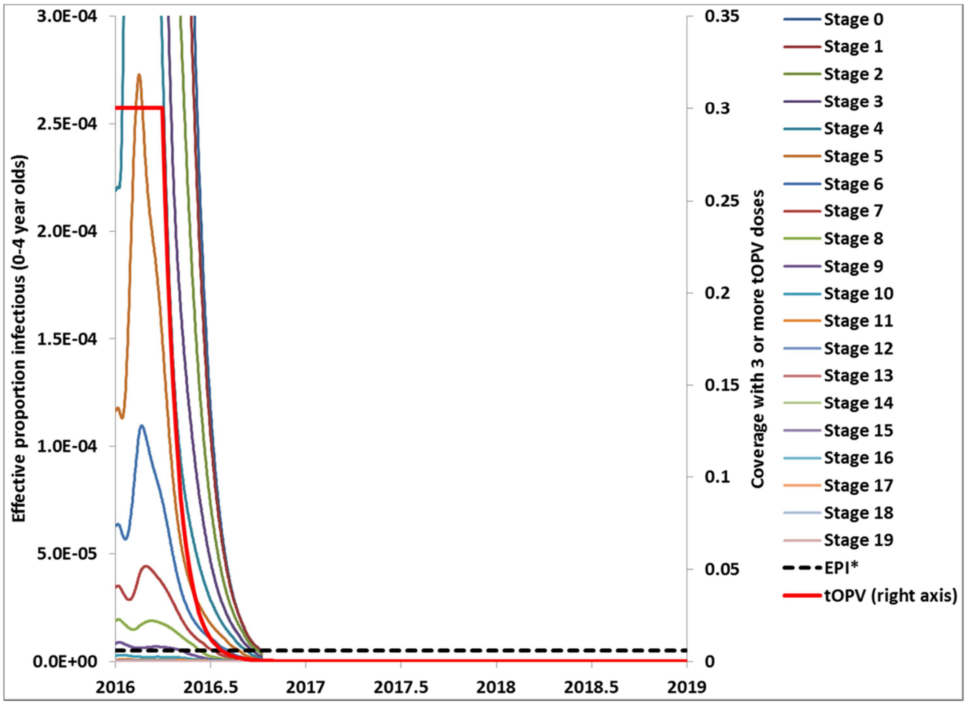

(d) Half-life of 30 days

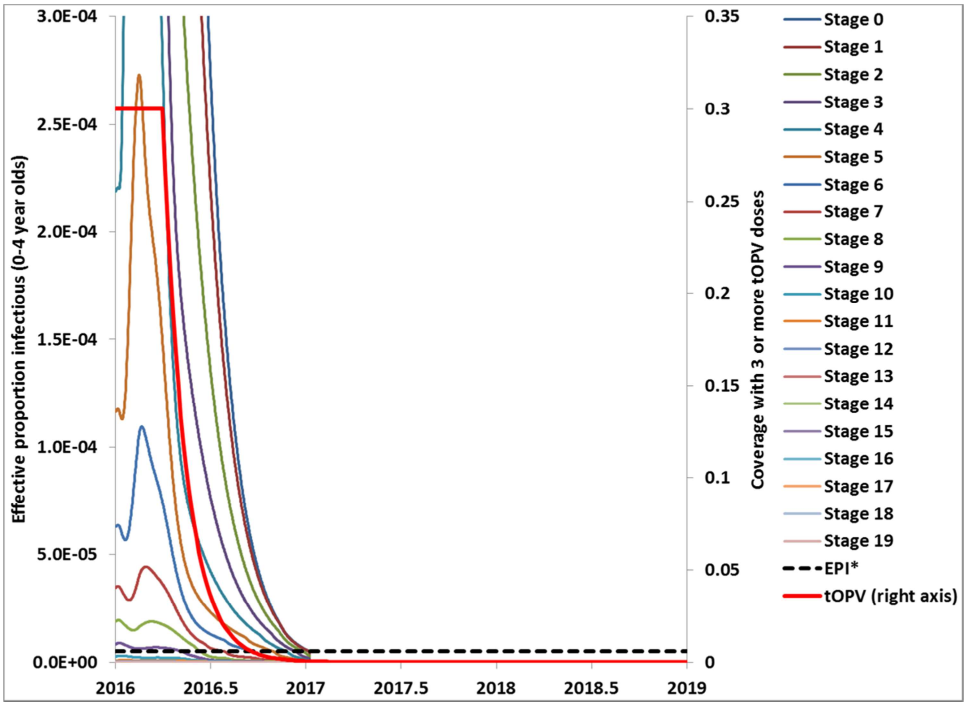

(e) Half-life of 40 days

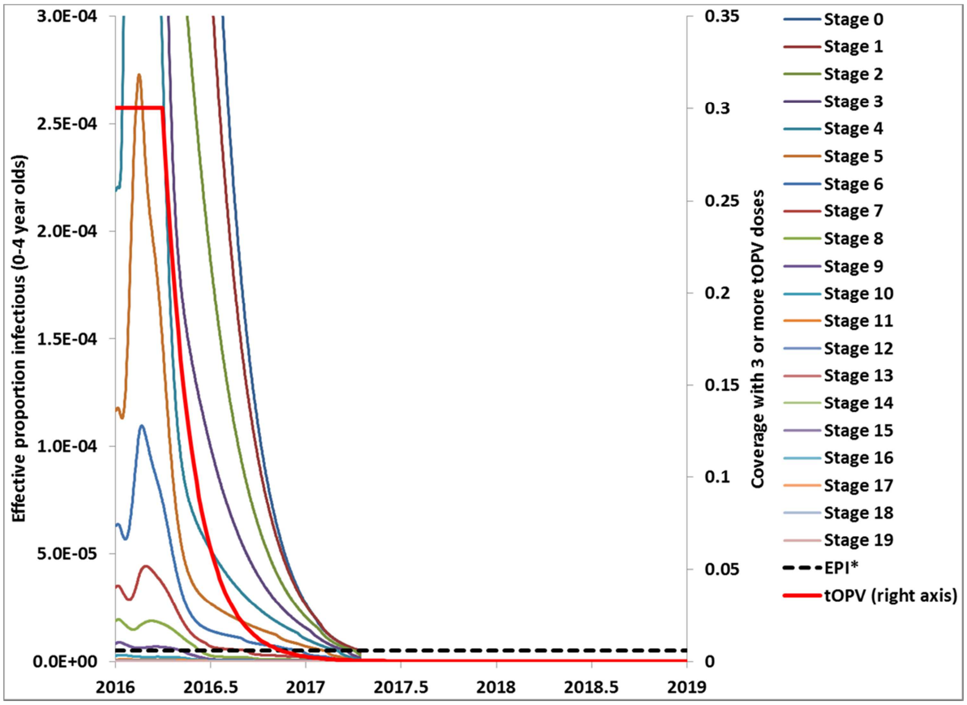

(f) Half-life of 50 days

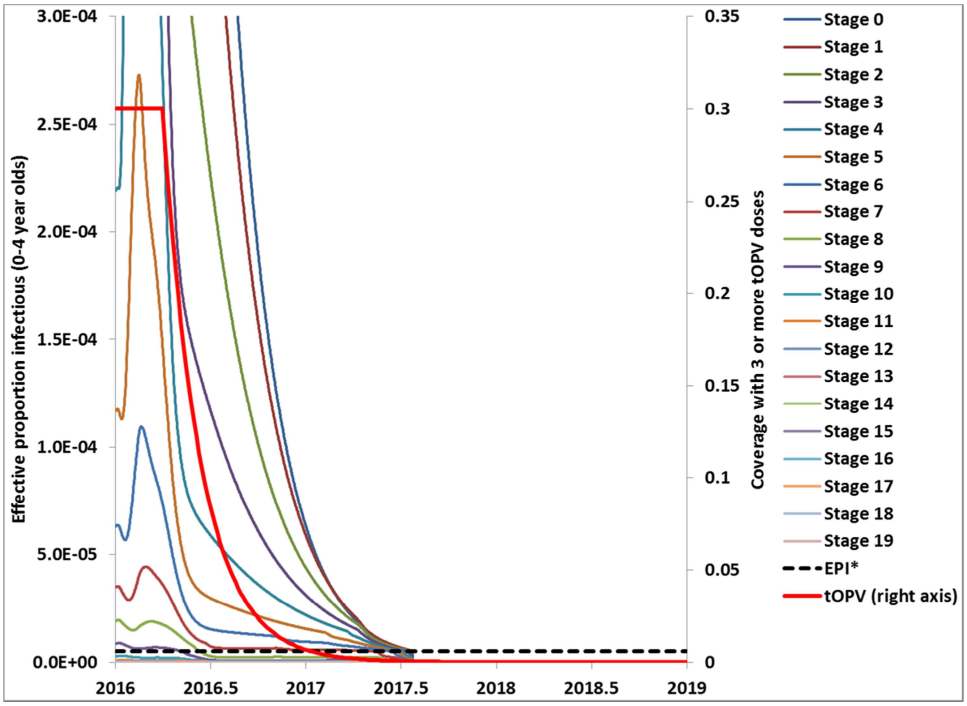

(g) Half-life of 58 days (shortest half-life that leads to a cVDPV2 outbreak)

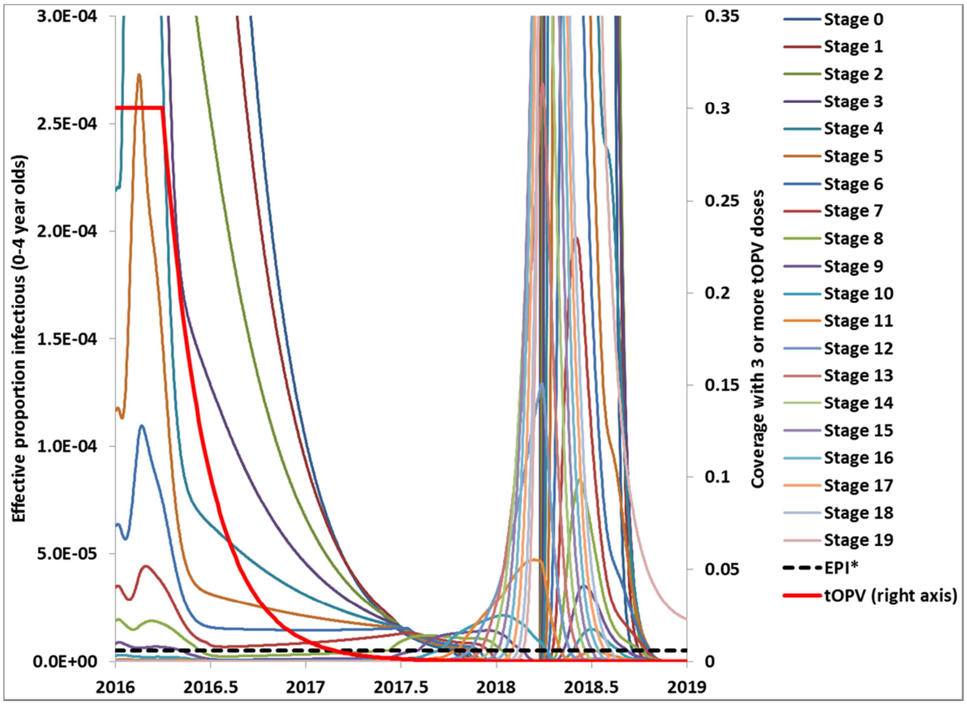

Supplement: Additional file 1: Figure S1. — Kinetics of prevalence and evolution of OPV2-derived viruses for the exponential decay pattern in the hypothetical population with a baseline R0 of WPV1 of 13. (EPI* = transmission threshold of effective proportion infectious below which the force-of infection becomes 0 in the differential equation-based model [15]). (PDF 1920 kb) [file 12879_2016_1537_MOESM1_ESM.pdf]
